# Association between CARD14 gene polymorphisms and psoriasis vulgaris in Hainan Han population based on exon sequencing: A case-control study

**DOI:** 10.1097/MD.0000000000030890

**Published:** 2022-10-07

**Authors:** Antonia Msafiri Makene, Jun-lin Liu

**Affiliations:** a Department of Dermatology and Venereology. The Second Affiliated Hospital of Hainan Medical University, Haikou, Hainan, China.

**Keywords:** CARD14 gene, psoriasis, haplotype, single nucleotide polymorphism

## Abstract

Psoriasis is a serious non-communicable, chronic immune-inflammatory mediated disease affecting about 125 million people worldwide. Its effects go beyond skin manifestation. Through genome-wide association studies, the caspase recruitment domain family member 14 (CARD14) gene and other gene variants have been implicated to have an association with Psoriasis, and as we move towards individualized therapy the discovery of single nucleotide polymorphism (SNP) is of great importance. This study aimed to determine whether the CARD14 gene is a susceptible gene for psoriasis vulgaris. In this study, 101 psoriasis patients and 79 healthy controls were subjected to exome sequencing. The CARD14 gene regions upstream and downstream of 1kb were sequenced. SNP-based association analysis and haplotype-based association analysis were performed in SNPs with minimum allele frequency (MAF) greater than 1%. Bioinformatic methods were used to predict the impact of risk loci on gene function. A total of 32 polymorphisms were identified in this study, of which 3 SNPs (1 in exon and 2 in intron) were susceptible to psoriasis (*P < *.05, OR = 0.19~0.53, 95%CI = 0.05~0.70). Bioinformatics analysis showed that rs144475004 located on the exon led to an amino acid change from aspartate to histidine. On the other hand, results of haplotype-based association analysis showed that 2 haplotypes (CARD14-1 and CARD14-2) were protective haplotypes of the disease (*P* < .05, OR = 0.18~0.38, 95%CI = 0.05~0.88), the frequencies in healthy controls and patients was 6.96% and 1.49%, respectively. CARD14 gene is associated with susceptibility to psoriasis vulgaris in the Hainan Han population.

## 1. Introduction

Psoriasis is a chronic, immune, and inflammatory disease with multiple genetic predispositions under certain environmental stimulants.^[1]^ Clinically, psoriasis can be divided into 4 types: vulgaris, arthritis, pustular, and erythrodermic. Psoriasis vulgaris is the most common type and accounts for about 80% to 90% of all psoriasis cases. This type is characterized by well demarcated erythematous plaques covered with silvery scales.^[2,3]^ The histopathology shows excessive keratinocyte proliferation and lymphocyte infiltration; precisely Th17, Th1 cells and inflammatory dendritic cells.^[4]^ World Health Organization has specified psoriasis as a serious non-communicable disease.^[5]^ Psoriasis effects extend beyond skin manifestations. Further studies have shown that up to 42% of psoriasis patients can develop psoriasis arthritis,^[6]^ of which will predominantly begin in the first decade after the initial symptoms of psoriasis.^[1]^ Other co-morbidities that are associated with psoriasis are hypertension, diabetes mellitus, cardiovascular diseases, tonsillitis, depression and anxiety.^[4,7]^ Recently, psoriasis has also been associated with a high baseline risk of lymphoproliferative diseases.^[8]^ About 125 million people worldwide have psoriasis;^[3]^ around 2% to 5% of affected individuals are of Western European descent and 0.1% to 0.3% are of Asian descent.^[9]^ Between 1987 and 2012 the prevalence of psoriasis in the Chinese population has risen from 0.12% to 0.47%.^[1,10]^

The etiopathogenesis of psoriasis is still a puzzle,^[11]^ which makes its treatment more challenging, but prior studies have shown that environment, immunity (innate and adaptive) in conjunction with genetics may lead to the development of psoriasis. Psoriasis vulgaris has been shown to mainly mediate through the TNF-IL23-Th17 axis.^[12]^

Early genetic studies such as linkage analysis paved the way for understanding the role of genetics in the pathogenesis of psoriasis.^[13]^ To date, advances in genetic research and methods such as genome-wide association studies, have aided in altering our understanding of complex genetic traits and diseases.^[14,15]^ Since the discovery of the psoriasis susceptibility locus 1 which accounts for 35% to 50% of psoriasis heritability and its allele Human Leucocyte Antigen-Cw06:02 (HLA-Cw06:02),^[16]^ now more than 65% of psoriasis heritability can be explained by genetics.^[7]^ Studies show that apart from sporadic cases, psoriasis also tends to run in families. Children of parents who are both affected with psoriasis carry about a 40% chance of acquiring the disease, as opposed to 14% if 1 parent is affected, and 6% if a sibling is affected. These findings are significant even as low rates of cases arise from the general population.^[11]^ Furthermore, this familial tendency was supported in twin studies which showed that identical twins bear a higher risk rate compared to fraternal twins.^[17]^ Through genome-wide association studies and other targeted candidate gene approaches,^[18]^ more than 80 psoriasis susceptibility loci have been identified. Likewise, millions of single nucleotide polymorphisms (SNPs) have been found at a genome-wide significance level (*P* < 5 × 10^-8^). Many of these SNPs are situated near genes involved in adaptive and/or innate immunity and skin barrier function pathways.^[14,16,19]^ Nuclear Factor kappa B (NF-κB) pathway is involved in the maintenance of inflammation in chronic psoriasis. The majority of genes of innate immunity belong to this pathway including the caspase recruitment domain family member 14 (CARD14) gene.^[11,18]^ Previous study has shown that the CARD14 gene, also known as Bimp2 or CARMA2, found at chromosome 17q25 in psoriasis susceptibility locus 2, is associated with psoriasis.^[20]^ In healthy skin, CARD14 is primarily abundant in placenta and keratinocytes of the basal layer of the epidermis, compared to psoriatic skin lesions that have increased levels of CARD14 in the upper layers of the epidermis and reduced CARD14 levels in the basal layer.^[21]^

Under certain environmental triggers overexpression of the CARD14 gene through its variants can either upregulate or downregulate the function of NF-κB leading to psoriasis.^[22,23]^ The stimulation process of NF-κB is achieved through the CARD- BCL10-MALT1 complex. It has been shown that basal NF-κB levels are important to preserve skin homeostasis.^[21]^ Prior genome studies have made discoveries on different SNPs relating them to certain genes responsible for psoriasis including the SNPs of the CARD14 gene.^[23–25]^ The discovery of SNPs aids in the understanding of the genetics of the human phenotypic variation and especially the genetics of complex diseases such as psoriasis.^[26]^ As we move to an era of individualized treatment, identifying SNPs associated with certain diseases is of great importance.^[27]^ Prior studies have been done to identify variants of the CARD14 gene in the Chinese population.^[24,25]^ However, due to environmental factors, population distribution and different research methods, the research results are still controversial.^[28,29]^ In this study we aim to investigate CARD14 gene polymorphism’s relationship with psoriasis vulgaris in the Southern Chinese Han ethnic group.

## 2. Materials and Methodology

### 2.1. Subjects

Hainan Han nationality 101 psoriasis vulgaris cases (65 males and 36 females, aged 18-82 years [45.31 ± 14.05]) were recruited from March 2018 to February 2020 at the dermatology clinic and ward of our hospital (Second Affiliated Hospital of Hainan Medical University). Each patient met the diagnostic criteria for psoriasis vulgaris.^[30]^ Other forms of psoriasis were not included. 79 healthy controls (47 males and 32 females, aged 21-78 [41.760 ± 12.860]) were recruited from the medical checkup center of the same hospital. Individuals with a family or personal history of psoriasis and those who were not Hainanese since their grandparents’ generation or who had a clear history of immigration from outside the island were excluded. There was no kinship or statistical difference in age and gender between the 2 groups *(P* > .05). This study followed the guidelines of the Helsinki Declaration and was approved by the medical ethics committee of the Second Affiliated Hospital of Hainan Medical University. The research subjects signed the informed consent before blood sample collection.

### 2.2. Instruments and reagents

See Table 1.

**Table 1:** Instruments and reagents.

Reagent	Equipment	Supplier
Agilent sureselect^XT^ custom Kit, SureSelect^XT^, Herculase^®^ II Fusion	ABI 2720	Thermo Fisher Scientific, MA, USA
	Agilent 2100	Agilent Technologies, CA, USA
NEBNext^®^ dsDNA	NanoDrop 2000	Thermo Fisher Scientific, MA, USA
	Illumina cBot	Illumina, CA, USA
Dynabeads^®^MyOne™	Invitrogen Qubit 3.0	Thermo Fisher Scientific, MA, USA
	Eppendorf 5810R	Eppendorf, Hamburg, Germany
Agencourt AMPure XP-PCR	Illumina Hiseq 2500	Illumina, CA, USA
Agencourt SPRIselect, Agencourt AMPure XP-PCR		Beckman Coulter, USA

### 2.3. Methodology

#### 1.2.3. procedure.

According to the manufacturer’s instructions, DNA was isolated from venous blood samples of patients and controls using the QIAmp DNA isolation Kit (QIAGEN, MD). The quality was determined by Nanodrop 2000/Qubit. OD260/280 = 1.8~2.0 was considered good quality. The integrity of Deoxyribonucleic Acid (DNA) was confirmed by agarose gel electrophoresis.

**Figure 1.**
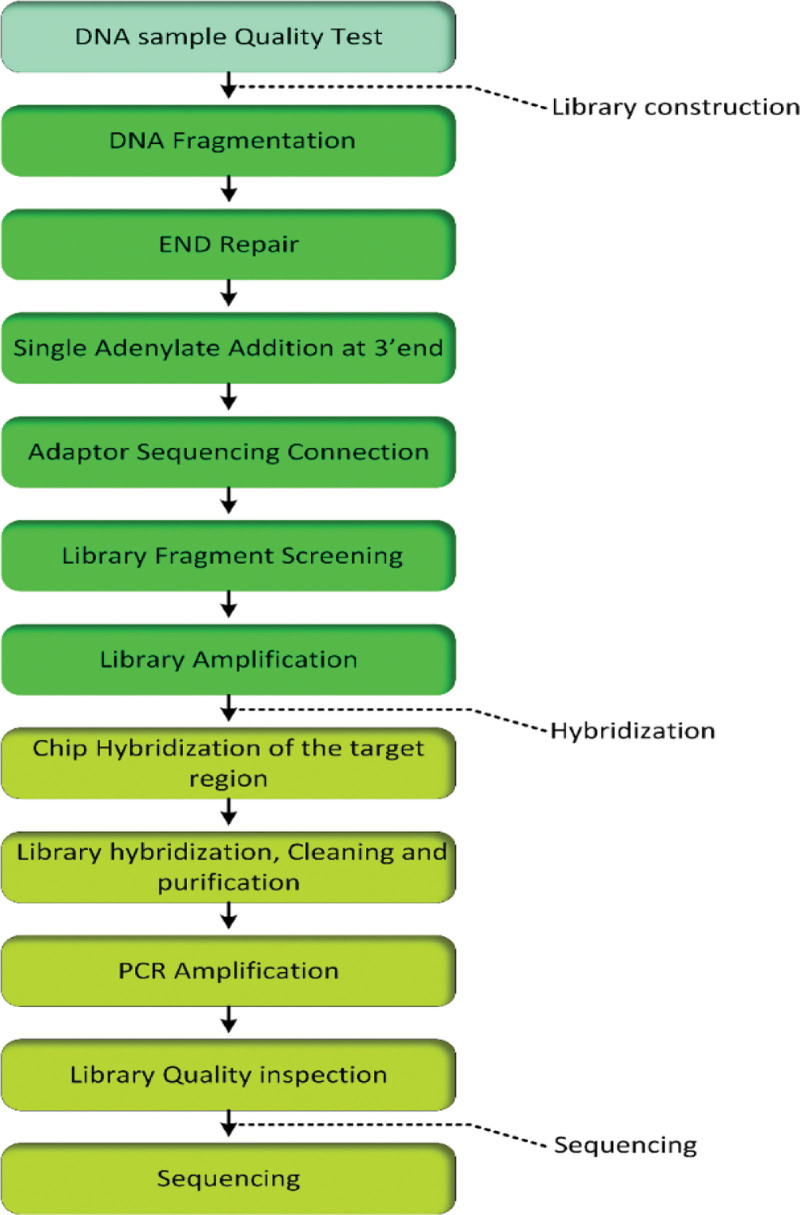
Exome sequencing flow chart.

Input DNA is converted to a sequencing library by fragmentation to 100-500bp fragments, the fragments underwent end repair by adding T4 DNA polymerase, then Agencourt AMpure XP magnetic beads were used to obtain the desired short fragment library Beads. Single adenylate A was added to the 3’end and ligation to platform-specific oligonucleotide adapters was done by T4 DNA ligase. The library was then purified and amplified ready for hybridization by Agilent sureselect^XT^ custom Kit, Cleaning and purification were done using Dynabeads^®^ MyOne™ Streptavidin T1. The library was again cleaned and quantified by Qubit 3.0 and the Agilent 2100 Bioanalyzer, respectively. The individual library fragments were clonally amplified by PCR; clusters of PCR colonies were then sequenced on the Illumina Hiseq2500 platform to obtain Fast SeQ data. The experimental steps are shown in Figure 1.

#### 2.2.3. analysis.

Hardy-Weinberg equilibrium test was performed for each SNP in the control group. The SNPs were screened by the following criteria: Hardy-Weinberg equilibrium test *P* > .05; Minimum allele frequency (MAF) > 0.01. The chi-square test and Fisher’s exact test were used for the screened SNPs, taking a single SNP as the unit, the association analysis was carried out according to 4 hypothetical genetic models; namely co-dominant model (with normal homozygous as the reference), dominant model (low-frequency allele as dominant), recessive model (low-frequency allele as recessive) and allele model. For each analysis, the false discovery rate and Bonferroni methods were used for correction. The above analyses were performed by PLINK 2.00 software. Haploview 4.2 software was used for haplotype-based association analysis.

#### 3.2.3. analysis.

Sorting intolerant from tolerant (SIFT) score software was used to predict whether amino acid changes affected the protein function. SIFT value of <.05, indicates that this variant severely affects protein function. SIFT score PRED software was used to predict pathogenicity, whereby D means Damaging and T means Tolerate.

## 3. Results

### 3.1. Demographic results

In our experiment were a total of 101 patients; 36 female and 65 male aged 18 to 82 years, mean ± sd: (45.310 ± 14.050), and 79 healthy controls 32 female and 47 male aged 21 to 78 years, mean ± sd: (41.760 ± 12.860). CARD14 gene exon and 1kb each of its upstream and downstream were sequenced. 32 SNPs were screened according to the above criteria for SNP and haplotype-based association analysis.

### 3.2. SNPs association analysis results

**Figure 2.**
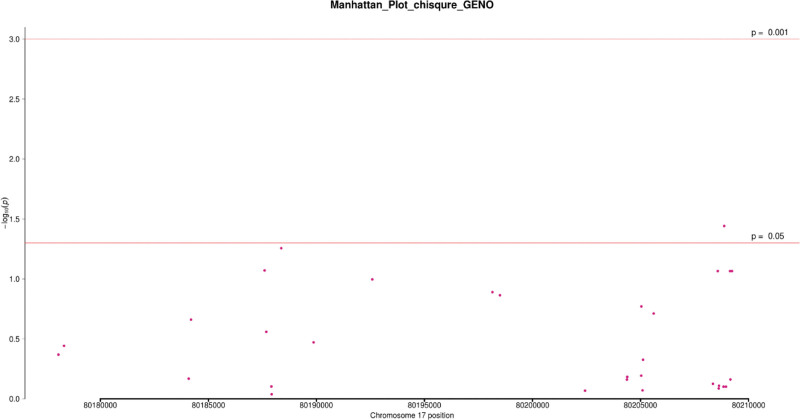
Manhattan plot of chi-square analysis based on Codominance model. Note: The abscissa is the chromosome position, and the ordinate is - log10 (P).

**Figure 3.**
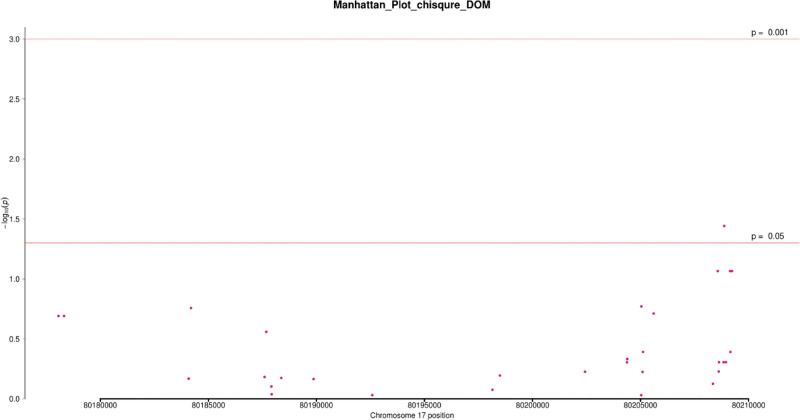
Manhattan plot of chi-square analysis based on dominant genetic model. Note: The abscissa is the chromosome position, and the ordinate is–log10 (p).

**Figure 4.**
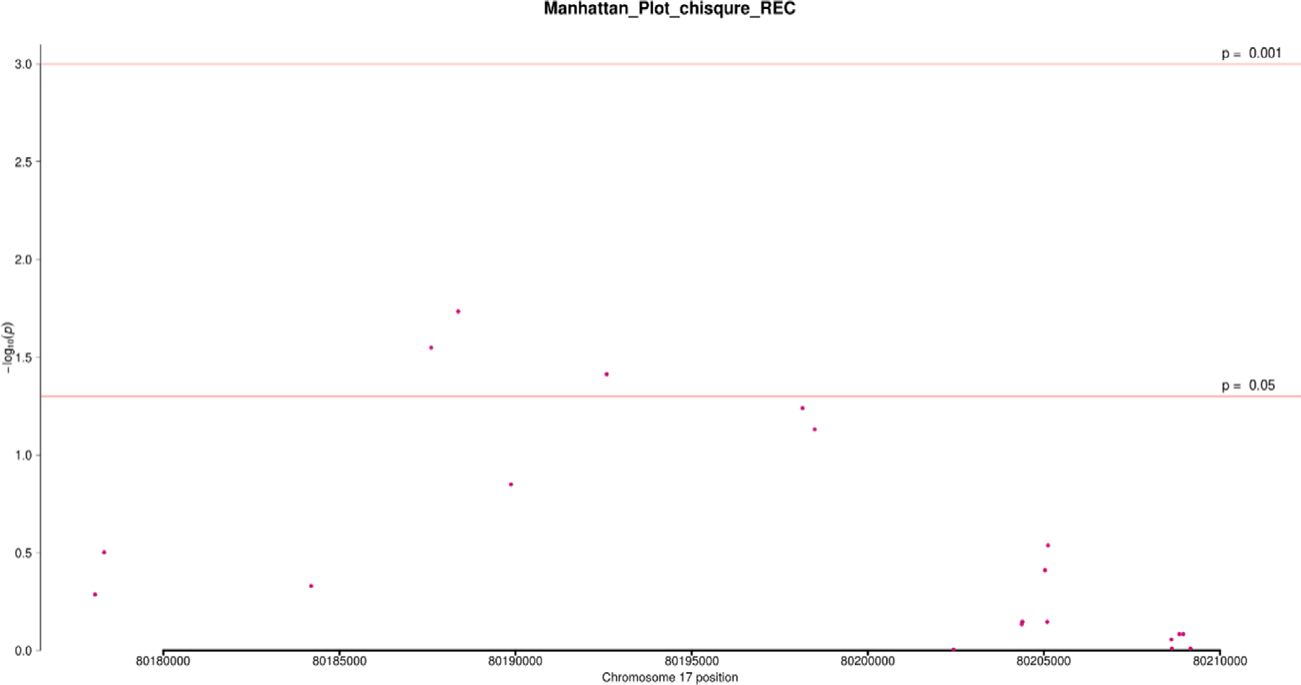
Manhattan plot of chi-square analysis based on recessive genetic model. Note: The abscissa is the chromosome position, and the ordinate is -log10 (p).

**Figure 5.**
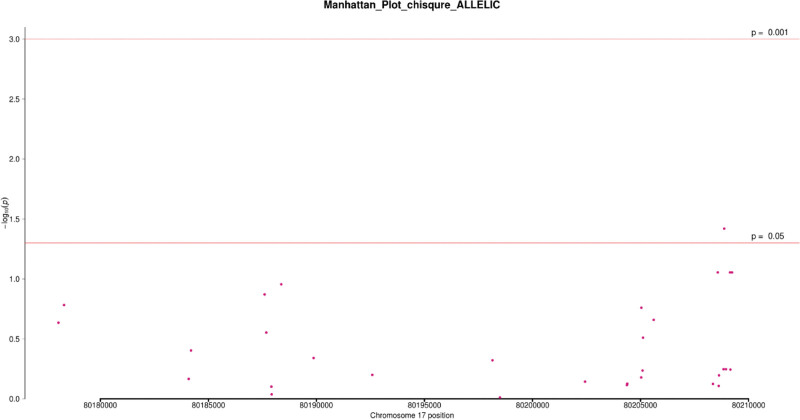
Manhattan plot of chi-square analysis based on allele genetic model. Note: The abscissa is the chromosome position, and the ordinate is -log10 (p).

In 101 patients and 79 controls, the Chi-square test was used to analyze the association of 32 SNPs that were screened under 4 genetic models (codominant, dominant, recessive, and allele). In at least one inheritance pattern, 3 SNPs had (*P* < .05 OR = 0.19~0.53, 95%CI = 0.05~0.70) (see Table 2) and (Figs. 2–5). Among the 3 SNPs, 1 was located in the exon region and 2 were located in the intron region. The genotype frequencies did not deviate from the Hardy–Weinberg equilibrium in controls (*P* > .05) (see Table 3). The results of the analysis using Fisher’s test were similar to the Chi-square test. The SNP rs144475004 G/C genotype found in the exon region led to amino acid changes from Aspartate to Histidine. Through bioinformatics analysis, this non-synonymous SNP (nsSNP) was predicted to be damaging with a SIFT score of 0.032.

**Table 2:** Association analysis results of the 3 SNPs with statistical differences (Chi-square test).

SNP	Model	Genotype	Cases (n)	Control (n)	Chi Score	OR (95%CI)	*P* value	FDR_BH Adjusted	Bonferroni adjusted
rs144475004	Co-dominant	CC	0	0	7.42		.01	0.19	0.21
		GC	3	11					
		GG	98	68					
	Dominant	CC,GC	3	11	7.42	0.19(0.05~0.70)	.01	0.21	0.21
		GG	98	68					
	Recessive	CC	0	0	NA	NA(NA~NA)	NA		
		GG,GC	101	79					
	Allele	C	3	11	7.12	0.20(0.06~0.74)	.01	0.25	0.25
		G	199	147					
rs4889836	Co-dominant	CC	4	12	8.07		.02	0.19	0.57
		TC	59	35					
		TT	38	32					
	Dominant	CC,TC	63	47	0.16	1.13(0.62~2.06)	.7	0.81	1
		TT	38	32					
	Recessive	CC	4	12	6.90	0.23(0.07~0.75)	.01	0.14	0.28
		TT,TC	97	67					
	Allele	C	67	59	0.68	0.83(0.54~1.29)	.41	0.85	1
		T	135	99					
rs4889989	Co-dominant	GG	22	22	3.88		.14	0.50	1
		CG	38	36					
		CC	41	21					
	Dominant	GG,CG	60	58	3.85	0.53(0.28~1.0)	.05	0.79	1
		CC	41	21					
	Recessive	GG	22	22	0.88	0.72(0.37~1.43)	.35	0.79	1
		CC,CG	79	57					
	Allele	G	82	80	3.61	0.67(0.44~1.01)	.06	0.80	1
		C	120	78					

CI = confidence interval, FDR = false discovery rate, OR = odd ratio, SNP single nucleotide polymorphism.

**Table 3:** Hardy-Weinberg equilibrium tests of 3 SNPs in control group.

SNP	*P*_HWE_
rs144475004	1
rs4889836	0.6
rs4889989	0.5

P_HWE_ = Hardy-Weinberg equilibrium value, SNP = single nucleotide polymorphism.

We additionally detected 2 intronic variants in our study; rs4889836 and rs4889989. A CARD14 gene variant rs4889836T/C genotype was significantly associated with psoriasis, the co-dominant and recessive genetic models (*P* = .02,.01) respectively. The recessive genetic model had an OR = 0.23, 95%CI = 0.07~0.75, with 3.96% and 15.19% frequencies in cases and control respectively. In addition, variant rs4889989 was found upstream in an intron area of the CARD14 gene. rs4889989 was significantly associated with psoriasis under the dominant genetic model (*P = *.05, OR = 0.53; 95% CI = 0.28~1.0). The genotype frequencies of these SNPs in cases and control are shown below (see Table 2) (Figs. 2–5).

### 3.3. based association analysis

Haplotype based association analyses revealed 2 haplotypes that were protective of the disease ([*P* = .01, .01], OR = 0.18~0.38, 95% CI: [0.05~0.88]). Frequencies in both cases and their healthy controls were 1.49% and 6.96% respectively (see Table 4).

**Table 4:** Results of haplotype analysis with statistical difference.

Hap	SNPS	Hap	Cases (%)	Control n (%)	*P* value	Estimate	Std error	Specificity	Sensitivity	Accuracy	*OR*(L95-U95)
CARD14-1	rs4889989, rs2289539, rs144475004	GTC	3(1.49%)	11(6.96%)	.01	-1.72	0.68	0.18	0.96	0.62	0.18 (0.05-0.68)
CARD14-2	rs144475004, rs4889990, rs3829612, rs532775553, rs3813064	CGCGC	3(1.49%)	11(6.96%)	.01	-1.61	0.68	0.2	0.95	0.60	0.38 (0.16-0.88)

Hap = haplotype, SNP = single nucleotide polymorphism, Std error = standard error, OR (L95-L95) Odd ration 95% confidence interval.

## 4. Discussion

In an earlier study, Jordan et al reported 23 CARD14 gene variants, mainly in patients with psoriasis vulgaris.^[22]^ Later, more genome-wide association studies reported more concerning the association of CARD14 gene variants and psoriasis vulgaris as well as other forms of psoriasis.^[1,29,31]^ In our study we found 4 CARD14 gene SNPs that are significantly associated with psoriasis vulgaris. The human genome comprises millions of SNPs.^[32]^ Clarifying this Barkur et al reported that SNPs can be divided into those that can change the encoded proteins (nonsynonymous) and the ones that take place in noncoding regions and therefore remain silent (synonymous). SNPs have the ability to influence gene expression or cause conformation and subcellular localization of mRNAs hence causing diseases.^[33]^ Research studies have shown that SNPs play an important role in the discovery of complex genetic diseases and certain cancers.^[34,35]^ These SNPs can be located in promoter, introns, exons and 3’-UTR and 5’-UTR regions within a gene structure.^[36]^ Previous studies have reported that exons 3 and 4 are the mutation hotspots of the CARD14 gene and that most of the variants found in these positions can lead to failure in sustaining the auto-inhibitory effect of NF- κB in the CARD- BCL10-MALT1 complex, leading to psoriasis.^[37,38]^ Exons are coding areas within a DNA or mRNA molecule. Studies have shown that there are numerous rare variants found within the exon region making the majority of the SNPs functional. The functional SNPs located in protein-coding areas, also known as regulatory SNPs, affect gene expression. Meanwhile, those that affect translation and splicing efficiency to enhance or inhibit alternative splicing (mRNA stability and protein function) are called structural RNA SNPs.^[39]^ In our cohort we detected a variant rs144475004G/C in exon 4 to be associated with psoriasis, in co-dominant, dominant and allele inheritance models *(P* = .01) in all models (see Table 2). Variant rs144475004 is a non-synonymous SNP that led to a substitution of base pairs from guanine to cytosine (G/C) leading to an amino acid change aspartate to histidine, a fact that could explain the risk susceptibility of this SNP to psoriasis. Moreover, our cohort bioinformatics analysis results, through SIFT pred test, showed the effect of rs144475004 was predicted to be damaging with a score of 0.032 indicating that this variant is deleterious. This could explain its pathogenicity. These results are consistent with those retrieved in previous studies.^[1,23,40]^ Huang et al also detected rs144475004G/C in the Chinese population with a MAF of 1.34% in psoriasis cases and 1.83% in East Asian healthy controls.^[1]^ Zhu et al (2016) reported an association between rs14475004 with psoriasis in Guangdong province, a Chinese population also situated in the upper part of southern China. In that 2016 study, this SNP was found in 3 cases frequency of 2.29% and one control subject frequency of 0.48%. Moreover, an assessment of the effects of this variant on protein function by Polyphen-2 revealed damaging results.^[41]^ In Europe Jordan et al reported that rs14475004 was a predisposing factor for generalized pustular psoriasis (GPP) with psoriasis vulgaris. Moreover, through NF- κB luciferase reporter assay, it was reported to upregulate the effect of NF-κB > 2 times as well as lead to stimulation of psoriasis-related genes.^[23]^ Later, a Japanese cohort also associated rs144475004 as a potential risk for GPP with psoriasis vulgaris, (*P* = .01; OR 8.62; 95% 1.75~42.4; power calculation of 0.609).^[28]^ Berki et al reported that this SNP leads to increased oligomerization of CARD14 gene and thereby increased association with GPP in Asian population (*P* = 8.4 × 10^ − 5^; OR = 6.4), but not associated with psoriasis vulgaris alone. In his previous study Mossner et al also reported that rs144475004 was a risk locus for Psoriasis vulgaris with palmoplantar pustular psoriasis in 2 Estonian patients.^[42]^ Qin et al also reported a MAF of 1.9% in psoriasis patients and 1.8% in controls in Chinese population.^[2]^ A recent large sample size study by Li et al did not find a significant relationship between the variant rs144475004 in GPP patients with psoriasis vulgaris (*P > *.05), and so concluded that the variant might not be the cause of disease in the Chinese population.^[43]^ On the other hand rs144475004 has also been associated with type I and IV pityriasis rubra pilaris (PRP).^[29,44]^ Yet, even though in this study rs144475004 was found to be damaging, haplotypes consisting of variant alleles from SNPs rs4889989, rs2289539 and rs144475004 from CARD14–1, GTC haplotype and SNPs rs144475004, rs4889990, rs3829612, rs532775553 and rs3813064 from CARD14-2, CGCGC haplotype showed a decreased risk of psoriasis (see Table 4). This indicates that a single SNP, apart from directly affecting the susceptibility to psoriasis, may also work in combination with other SNPs to alter the risk susceptibility to psoriasis vulgaris as a cumulative effect. Therefore, this effect can be inconsistent with the effect produced by a single SNP alone, as seen with rs144475004 in our study. Moreover, these findings could also be attributed to the small sample size of our study, and therefore these results will need to be further validated in large sample size studies.

Interestingly, even though we aimed at screening the exons of the CARD14 gene, 2 intronic SNPs in the range of 1kb upstream and downstream that related to the disease were also detected in our study. Introns are the non-protein-coding areas of a DNA sequence within a gene or a corresponding sequence in the unprocessed RNA transcript. The function of most intronic SNPs’ is unknown, but recently genome-wide association study has detected more functional SNPs.^[45]^ Some studies have reported that introns play a part in increasing the efficiency of mRNA translation and also increasing mRNA content via transcription, export, and stability within mammals.^[46]^ Variants in an intron, have been associated with more than 75 genetic diseases, and these intronic variants currently can be detected through RNA sequencing analysis.^[35]^ In our study a variant rs4889836T/C located in an intron region, was significantly associated with psoriasis, with the co-dominant and recessive inheritance pattern (*P* = .02, .01). The variant’s recessive model frequencies were 3.96% in cases and 15.19% in controls. The results of the association for this SNP with psoriasis susceptibility in the Chinese population are being reported for the first time, hence further studies are needed to confirm the functions of this SNP. Nevertheless, this variant was previously found in psoriasis patients of the northern Spanish population in intron 17 with a mean allele frequency of 0.49. In which case bioinformatics analysis results predicted it to be benign, showing that this variant might only be a risk locus for psoriasis and not pathogenic.^[25]^

We also detected one new SNP, rs4889989. A variant rs4889989 was found within 1kb near the promoter upstream of an intron region. Our results revealed that this SNP is significantly associated with psoriasis vulgaris (*P* = .05), the dominant genotype GC of this variant was found in 73.42% of cases and 59.41% of controls. A protective association was observed on the CARD14-1, GTC haplotype (rs4889989, rs2289539, rs144475004) (*P = *.01). These findings show that this SNP could be a common variant and that it probably requires other cofactors to be able to cause disease. This SNP has not been reported in previous studies.^[47]^

On the other hand, among the detected SNPs a common variant rs11652075C/T was detected in our study, but there was no significant association between this SNP genotype and allele frequencies with risk for psoriasis vulgaris in this study (*P* = .1, .5) respectively. Nonetheless, a prior study reported the association between a common variant rs11652075 with psoriasis in the European and Chinese Han populations.^[23]^ A small meta-analysis done by Jordan et al from 6 European cohorts revealed 4 missense variants (rs11652075 included) to be associated with psoriasis, with c.2458C being a risk allele, their fixed and random effects (*P* = 2.13 × 10^-6^, .031). The study further found that this SNP can stimulate NF-κB leading to psoriasis. This finding was pronounced when this SNP was conditioned on psoriasis susceptibility locus 1.^[23]^ Additionally the same risk allele, was also associated with psoriasis (*P* = .03) in Asians.^[24,41]^ These results were further validated by Shi et al meta-analysis.^[48]^ Meanwhile, Judith et al found a couple of CARD14 gene variants (rs117918077, rs2066964, rs28674001, and rs11652075) in a Hungarian patient with childhood-onset PRP that worsened in adulthood, whose lesions were unresponsive to retinoic acid treatment. The 4 SNPs found in this PRP patient were found to increase the activation of NF- κB.^[49]^ Despite previous studies demonstrating the association between rs11652075 with psoriasis, Sugiura et al reported similar results as in our study. The report showed that a combination of rs11652075 with Down syndrome might be a reason for early-onset psoriasis arthritis, but alone might not be a risk for psoriasis vulgaris.^[50]^ Other variants rs4889990 and 2066964 were also previously documented SNPs with risk susceptibility to psoriasis,^[23,25]^ but the opposite is true for our study (*P* = .4, .3) respectively.

It should be noted that the inconsistency of the current research results may be related to the small sample size, race, and environment of the study group, diversity of gene expression, or experimental method used. In the future, the 4 SNPs can be used as candidate loci to conduct in-depth analysis and functional research of susceptibility loci in larger sample sizes and populations of different races. Moreover, protein function analysis was not performed for some of the SNPs, hence providing room for further studies. Additional analysis and functional study of susceptibility loci will be needed to comprehensively and accurately reveal the relationship between CARD14 gene polymorphisms and psoriasis vulgaris and to further explore the impact of CARD14 gene SNPs on the pathogenesis and severity of psoriasis and the role in the stimulation of NF-κB that may lead to psoriasis vulgaris.

## 5. Conclusion

Our study was designed to assess the association between CARD14 gene polymorphisms and psoriasis vulgaris in the Hainan Han population. Our main findings can be summarized as follows; 3 SNPs were associated with psoriasis one from the exon area and 2 from the intron area. The 2 intronic variants are being reported for the first time in our population. The exonic SNP led to protein substitution which could explain its pathogenicity as seen via the bioinformatics test and score. Haplotype findings revealed a protective association with the disease. This indicated that the disease may be affected differently by SNPs in a cumulative effect than by a single SNP and that there may be multifactorial reasons for disease causation and development.

The findings of the 3 CARD14 variants, 2 known and one new variant, from this study, make several contributions to the current literature and gene database; all of which have established the risk association with psoriasis vulgaris in the Hainan Han population.

The authors would like to thank the National Natural Science Foundation of China (81860551) for funding this study.

## Author contributions

**Conceptualization:** Jun Lin Liu, Antonia Msafiri Makene.

**Data curation:** Jun Lin Liu, Antonia Msafiri Makene.

**Formal analysis:** Jun Lin Liu, Antonia Msafiri Makene.

**Funding acquisition:** Jun Lin Liu.

**Investigation:** Jun Lin Liu, Antonia Msafiri Makene.

**Methodology:** Jun Lin Liu, Antonia Msafiri Makene.

**Project administration:** Jun Lin Liu.

**Resources:** Jun Lin Liu.

**Software:** Jun Lin Liu.

**Supervision:** Jun Lin Liu.

**Validation:** Jun Lin Liu.

**Visualization:** Antonia Msafiri Makene.

**Writing – original draft:** Antonia Msafiri Makene.

**Writing – review & editing:** Jun Lin Liu, Antonia Msafiri Makene
